# Supplementary material for: Plasma membrane phosphatidylinositol (4,5)-bisphosphate is critical for determination of epithelial characteristics
Source: Nat Commun. 2022 May 9;13:2347. doi: 10.1038/s41467-022-30061-9 (PMC9085759; doi:10.1038/s41467-022-30061-9)
Supplement: Supplementary file 1 — Supplementary Information [file 41467_2022_30061_MOESM1_ESM.pdf]

SUPPLEMENTARY INFORMATION

Plasma membrane phosphatidylinositol (4,5)-bisphosphate is critical for determination of epithelial characteristics

Kaori Kanemaru[1,10], Makoto Shimozawa[2,10], Manabu Kitamata[2], Rikuto Furuishi[1], Hinako Kayano[2], Yui Sukawa[2], Yuuki Chiba[2], Takatsugu Fukuyama[2], Junya Hasegawa[3,4], Hiroki Nakanishi[5,6], Takuma Kishimoto[7], Kazuya Tsujita[8,9], Kazuma Tanaka[7], Toshiki Itoh[8, 9], Junko Sasaki[3,4], Takehiko Sasaki[3,4], Kiyoko Fukami[2*], Yoshikazu Nakamura[1*]

[1] Department of Applied Biological Science, Faculty of Science and Technology, Tokyo University of Science, Noda, Chiba 278-8510 Japan

[2] Laboratory of Genome and Biosignals, School of Life Sciences, Tokyo University of Pharmacy and Life Sciences, Hachioji, Tokyo 192-0392 Japan

[3] Department of Biochemical Pathophysiology, Medical Research Institute, Tokyo Medical and Dental University, Bunkyo-ku, Tokyo 113-8510, Japan

[4] Department of Lipid Biology, Graduate School of Medical and Dental Sciences, Tokyo Medical and Dental University, Bunkyo-ku, Tokyo 113-8510, Japan

[5] Research Center for Biosignal, Akita University, Akita-city, Akita 010-8543, Japan.

[6] Lipidome Lab Co., Ltd, Akita-city, Akita 010-0825, Japan

[7] Division of Molecular Interaction, Institute for Genetic Medicine, Hokkaido University Graduate School of Life Science, Sapporo, Hokkaido 060-0815, Japan

[8] Biosignal Research Center, Kobe University, Kobe, Hyogo 657-8501, Japan

[9] Division of Membrane Biology, Department of Biochemistry and Molecular Biology, Kobe University Graduate School of Medicine, Kobe, Hyogo 650-0017, Japan

[10] These authors contributed equally: Kaori Kanemaru, Makoto Shimozawa

*Correspondence to Yoshikazu Nakamura (ynakamur@rs.tus.ac.jp) or Kiyoko Fukami (kfukami@toyaku.ac.jp)

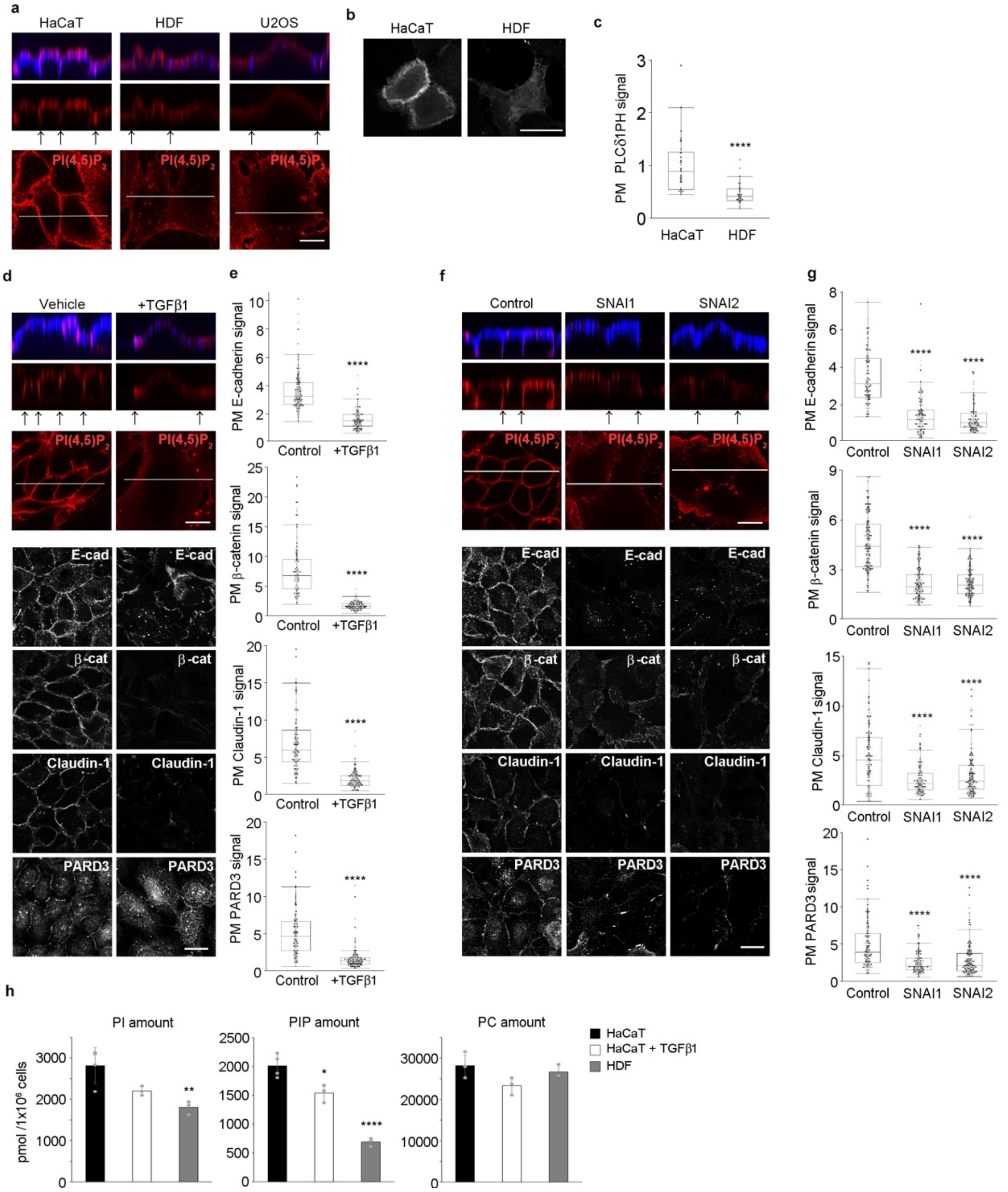

Supplementary Figure 1. Epithelial cells have higher amount of PI(4,5)P_2 than non-epithelial cells

a Immunofluorescence detection of PI(4,5)P_2 in HaCaT, HDF, and U2OS cells. The xz sections are shown. Lines indicate the position where xz sections were taken. Arrows indicate plasma membrane (PM). The PM was stained with WGA (blue staining). Results shown are representative of two independent experiments. **b, c** Localization of GFP-fused PH domain of PLCδ1 in HaCaT and HDF cells (b). Quantification of apicolateral PM signals of GFP-fused PH domain of PLCδ1 (c). In total, 34 HaCaT cells and 36 HDF cells were examined over two independent experiments (c). **d-g** Immunofluorescence detection

of $PI(4,5)P_2$, E-cadherin (E-cad), β-catenin (β-cat), claudin-1, and PARD3 in HaCaT cells treated with or without TGFβ1 for 72 h (d) and HaCaT cells overexpressing SNAI1 or SNAI2 (f). Lines indicate the position where xz sections were taken. Arrows indicate PM. The PM was stained with WGA (blue staining). Images were taken from different fields of view. Quantification of the PM signals of E-cadherin, β-catenin, claudin-1, and PARD3 (e, g). In total, 145 control and 101 TGFβ1-treated cells were examined over two independent experiments for E-cadherin. In total, 104 control and 106 TGFβ1-treated cells were examined over two independent experiments for β-catenin. In total, 109 control and 107 TGFβ1-treated cells were examined over two independent experiments for claudin-1. In total, 106 control and 114 TGFβ1-treated cells were examined over two independent experiments for PARD3 (e). In total, 104 control, 106 SNAI1-, and 105 SNAI2-expressing cells were examined over two independent experiments for E-cadherin. In total, 134 control, 106 SNAI1-, and 105 SNAI2-expressing cells were examined over two independent experiments for β-catenin. In total, 106 control, 103 SNAI1-, and 117 SNAI2-expressing cells were examined over two independent experiments for claudin-1. 108 control, 103 SNAI1-, and 119 SNAI2-expressing cells were examined over two independent experiments for PARD3 (g). **h** Amounts of PI, PIP, and PC in untreated HaCaT, TGFβ1-treated HaCaT, and HDF cells were determined using mass spectrometry. The levels are reported as the amount per $1{\times}10^6$ cells. Data are represented as mean ± SD. $N = 4$ for untreated HaCaT cells, and $N = 3$ for TGFβ1-treated HaCaT and HDF cells. $^{**}p = 0.0085$, $^{*}p = 0.0145$ (versus HaCaT cells) (h). The box plots are presented with the elements: center line, median; box limits, Q1 and Q3; whiskers, 1.5× interquartile range. Outliers are also shown. Individual data points are displayed [gray points, data from the first experiments; black points, data from the second experiments (c, e, g)]. Significance was tested using one-way ANOVA with Tukey-Kramer's post hoc test (g, h) and the two-sided Welch's t-test (c, e). $^{****}p < 0.0001$ [versus HaCaT cells (c and h) and control cells (e and g)]. Scale bar = 20 μm. Source data are provided as a Source Data file.

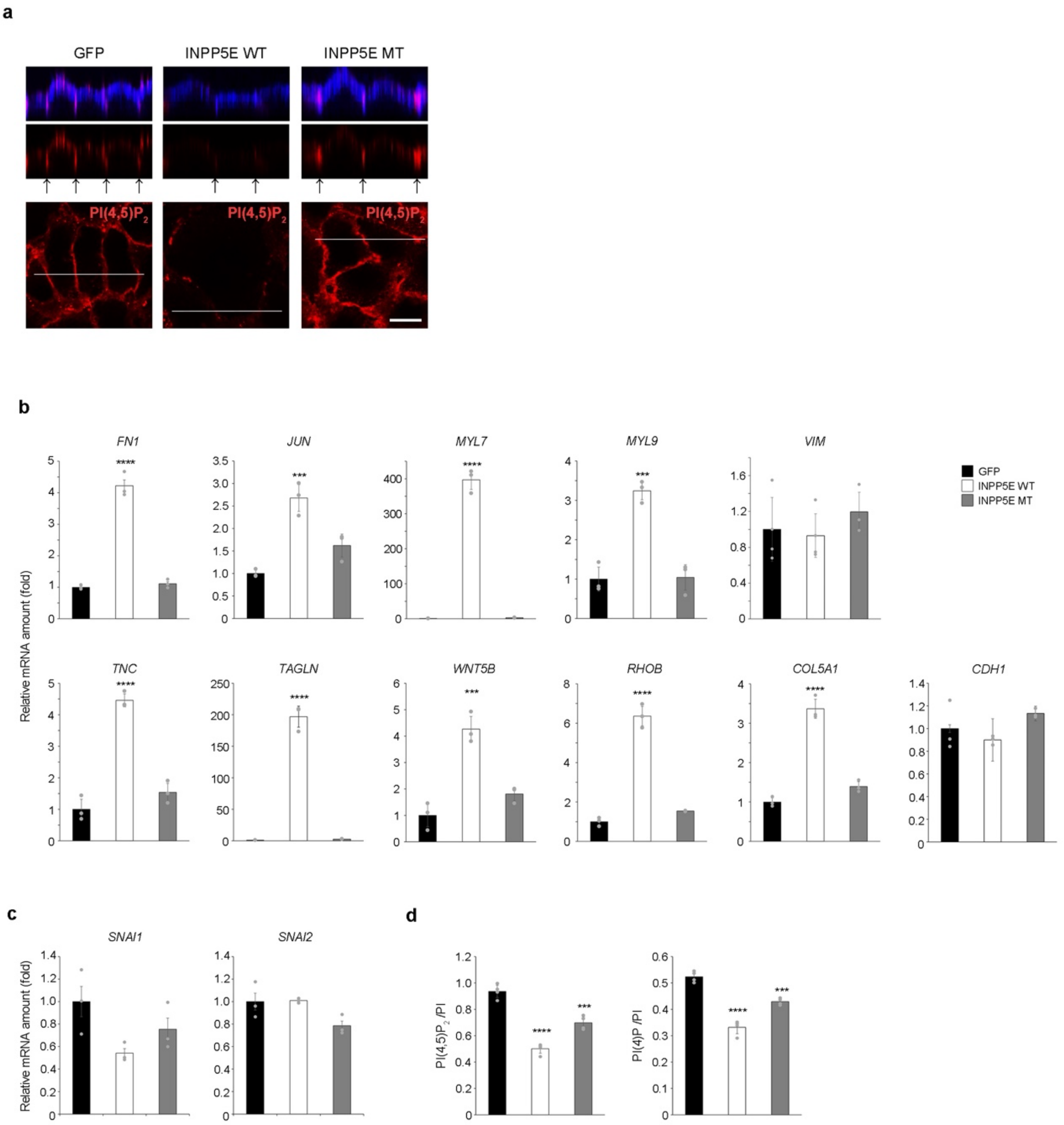

Supplementary Figure 2. Plasma membrane PI(4,5)P2 depletion induces expression of EMT core genes

a Immunofluorescence detection of PI(4,5)P2 in GFP-, Lyn-INPP5Ewt-GFP (INPP5E WT)-, and Lyn-INPP5Emt-GFP (INPP5E MT)-expressing HaCaT cells. Lines indicate the position where xz sections were taken. Arrows indicate PM. The plasma membrane (PM) was stained with WGA (blue staining). Results shown are representative of two independent experiments. Scale bar = 20 μm. **b, c** *FN1* (b), *JUN* (b), *MYL7* (b), *MYL9* (b), *VIM* (b), *TNC* (b), *TAGLN* (b), *WNT5B* (b), *RHOB* (b), *COL5A1* (b), *CDH1* (b), *SNAI1* (c), and *SNAI2* (c) mRNA expression levels in GFP-, INPP5E WT-, and INPP5E MT-expressing HaCaT cells were determined using real-time RT-PCR. Data are represented as mean ± SD. *N* = 3 for each group except GFP- and INPP5E WT-expressing cells for

VIM (*N*=4). ***p = 0.0008 (*JUN*), 0.0006 (*MYL9*), and 0.0004 (*WNT5B*) (versus GFP-expressing cells). **d** The amount of PI(4,5)P_2 and PI(4)P in GFP-, INPP5E WT-, and INPP5E MT-expressing HaCaT cells was determined using mass spectrometry. The levels are reported as the ratio of PI(4,5)P_2 and PI(4)P to PI. $N = 4$ for each group. Data are represented as mean ± SD. ***p = 0.0002 [PI(4,5)P_2] and 0.0004 [PI(4)P] (versus GFP-expressing cells). Significance was tested using one-way ANOVA with Tukey-Kramer's post hoc test. ****$p < 0.0001$ (versus GFP-expressing cells). Source data are provided as a Source Data file.

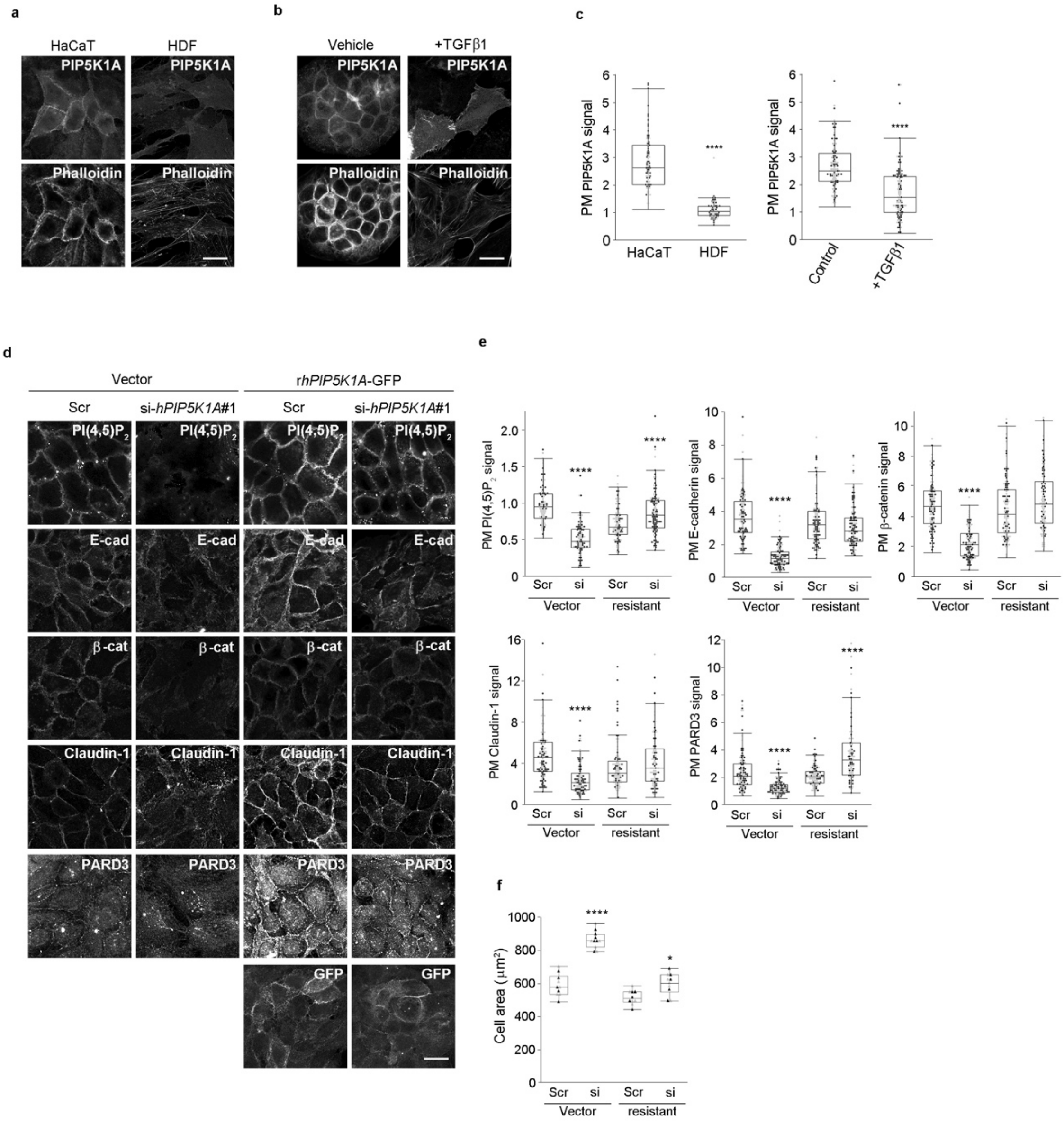

Supplementary Figure 3. PIP5K1A is involved in the maintenance of epithelial characteristics in HaCaT cells

a-c Detection of hPIP5K1A-GFP in HaCaT cells treated with or without TGFβ1 for 72 h (a, b) and HDF (a). Quantification of plasma membrane (PM) signals of hPIP5K1A-GFP (c). In total, 103 HaCaT and 77 HDF cells were examined over two independent experiments. In total, 122 control and 102 TGFβ1-treated cells were examined over two independent experiments (c). **d-e** Detection of PI(4,5)P_2, E-cadherin (E-cad), β-catenin (β-cat), claudin-1, PARD3, and siRNA-resistant hPIP5K1A-GFP (GFP) in empty vector (Vector) or siRNA-resistant hPIP5K1A (rhPIP5K1A-GFP or resistant)-expressing HaCaT cells treated with scrambled (Scr) or *hPIP5K1A*-targeting (PIP5K1A#1 or si) siRNAs. Images were taken from different fields of view. Quantification of the PM

signals of $PI(4,5)P_2$, E-cadherin, β-catenin, claudin-1, and PARD3 (e). In total, 104 vector/Scr, 105 vector/si, 102 resistant/Scr, and 115 resistant/si cells were examined over two independent experiments for $PI(4,5)P_2$. In total, 117 vector/Scr, 111 vector/si, 112 resistant/Scr, and 110 resistant/si cells were examined over two independent experiments for E-cadherin. In total, 120 vector/Scr, 102 vector/si, 119 resistant/Scr, and 111 resistant/si cells were examined over two independent experiments for β-catenin. In total, 119 vector/Scr, 106 vector/si, 105 resistant/Scr, and 100 resistant/si cells were examined over two independent experiments for claudin-1. In total, 119 vector/Scr, 106 vector/si, 105 resistant/Scr, and 100 resistant/si cells were examined over two independent experiments for PARD3 (e). **f** Cell areas of empty vector or siRNA-resistant hPIP5K1A-expressing HaCaT cells treated with scrambled (Scr) or *hPIP5K1A*-targeting (PIP5K1A#1) siRNAs. The average cell area was calculated by analyzing 10 distinct fields of view for vector/Scr, resistant/Scr, and resistant/si cells or 11 distinct fields of view for vector/si cells over two independent experiments. *$p = 0.02$ (versus siRNA-resistant hPIP5K1A-expressing HaCaT cells treated with scrambled siRNA). The box plots are presented with the elements: center line, median; box limits, Q1 and Q3; whiskers, 1.5× interquartile range. Outliers are also shown. Individual data points are displayed (gray points, data from the first experiments; black points, data from the second experiments). Significance was tested using one-way ANOVA with Tukey-Kramer's post hoc test (e, f) and the two-sided Welch's t-test (c). ****$p < 0.0001$ [versus HaCaT cells (c), HaCaT cells treated with scrambled siRNA in each condition (e and f)]. Scale bar = 20 μm. Source data are provided as a Source Data file.

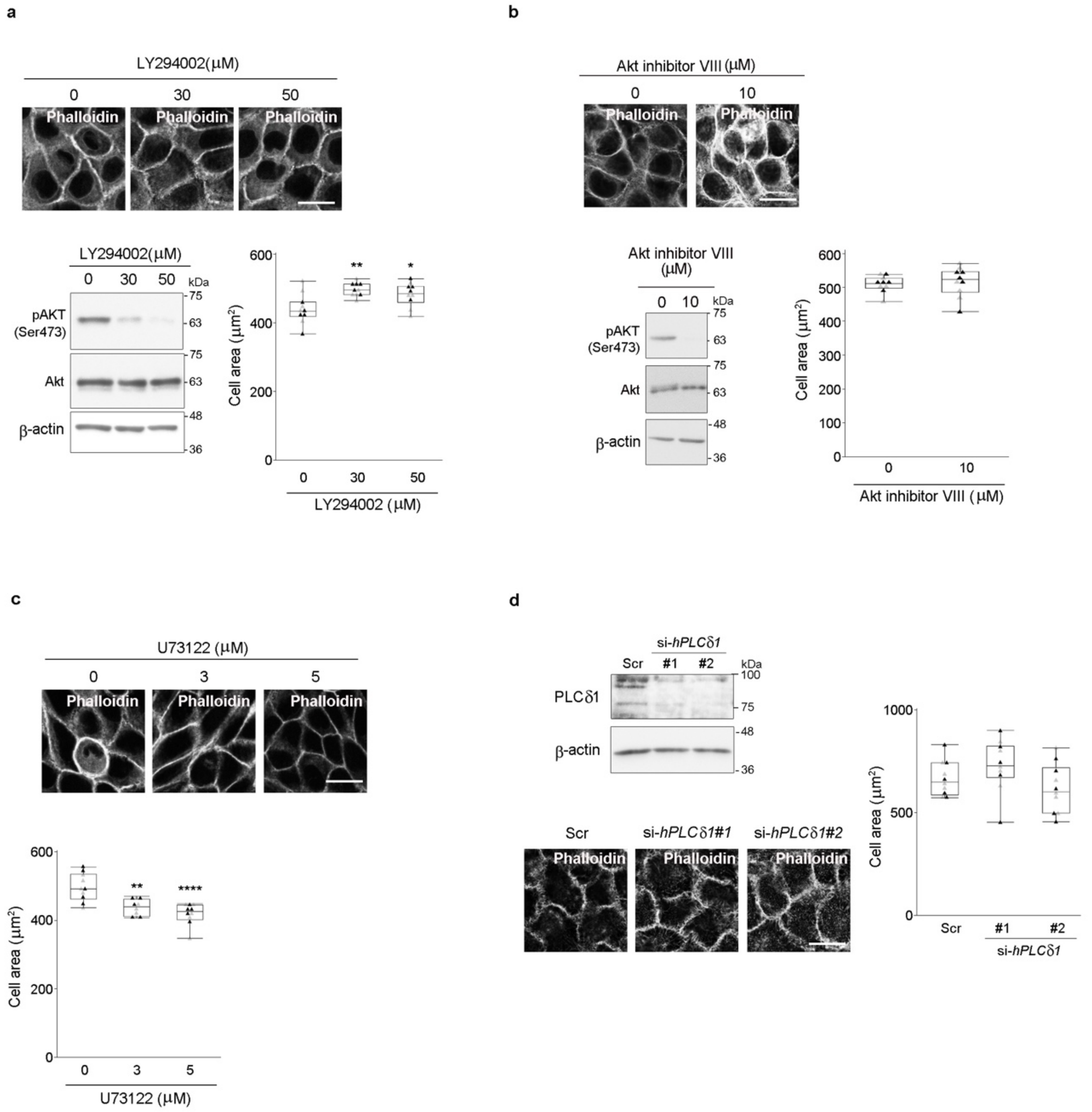

Supplementary Figure 4. Inhibition of production of PI(4,5)P$_2$-derived signaling molecules does not affect epithelial characteristics

a, b Detection of F-actin (phalloidin) in HaCaT cells treated with LY294002 (a) or AKT inhibitor VIII (b) at the indicated concentrations. Immunoblotting of Akt and phosphorylated Akt (pAkt) in HaCaT cells treated with LY294002 (a) or AKT inhibitor VIII (b). β-actin was used as the loading control. Immunoblot data shown are representative of two independent experiments with similar results. The cell area of HaCaT cells treated with LY294002 (a) or AKT inhibitor VIII (b) is also shown. **p = 0.002, *p = 0.0225 (versus untreated cells). **c, d** Detection of F-actin (phalloidin) in HaCaT cells treated with U73122 (c) or scrambled (Scr) or *PLCδ1*-targeting (PLCδ1#1 and PLCδ1#2) siRNAs. The cell area of HaCaT cells treated with U73122 or siRNAs is

also shown. ****$p < 0.0001$, **$p = 0.0013$ (versus untreated cells). Immunoblotting of PLCδ1 in HaCaT cells treated with scrambled (Scr) or *PLCδ1*-targeting (PLCδ1#1 and PLCδ1#2) siRNAs. β-actin was used as loading control (d). Immunoblot data shown are representative of two independent experiments with similar results. The average cell area was calculated by analyzing 10 distinct fields of view over two independent experiments. The box plots are presented with the elements: center line, median; box limits, Q1 and Q3; whiskers, 1.5× interquartile range. Outliers are also shown. Scale bar = 20 μm. Individual data points are displayed (gray points, data from the first experiments; black points, data from the second experiments). Significance was tested using one-way ANOVA with Tukey-Kramer's post hoc test. Source data are provided as a Source Data file.

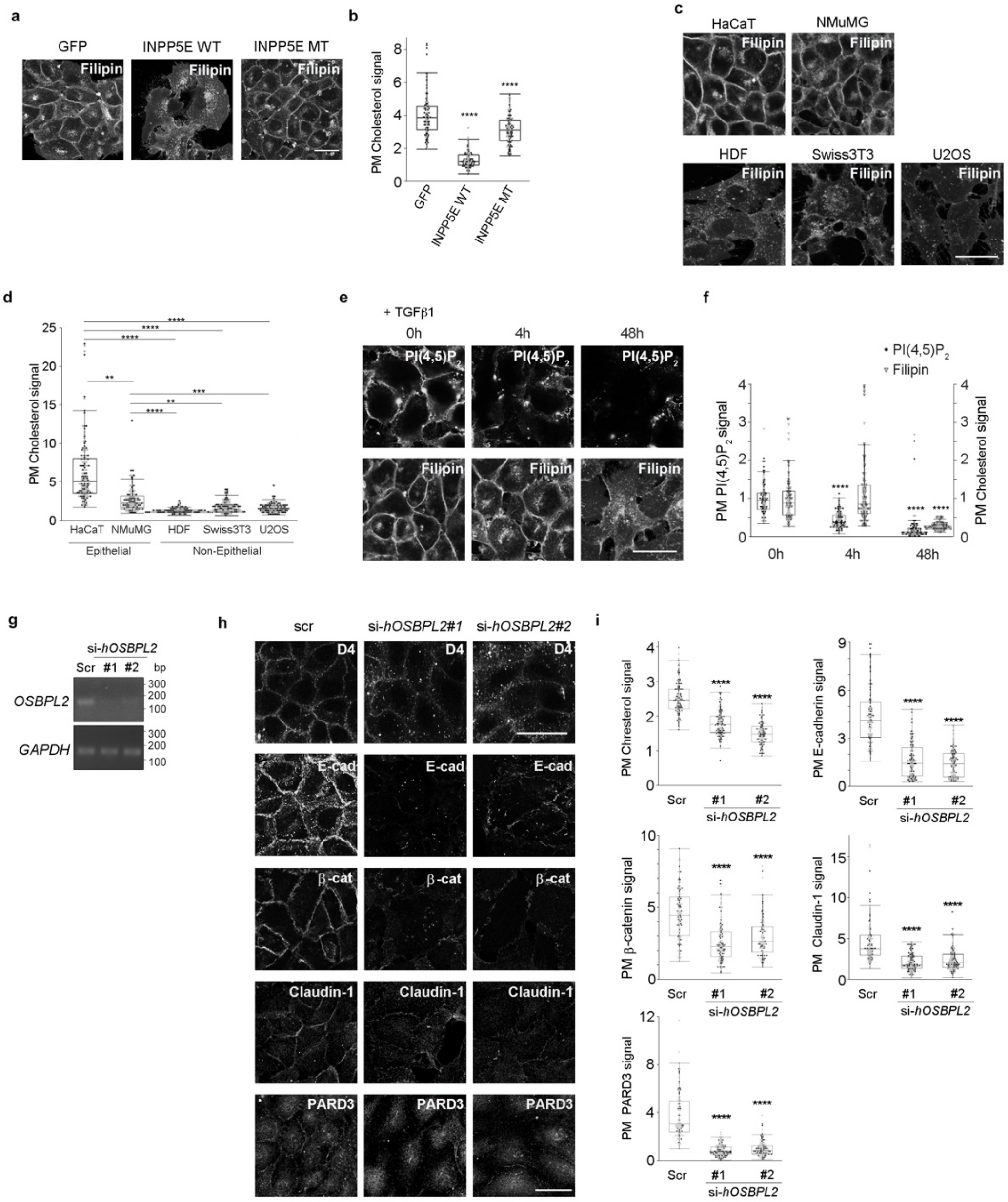

Supplementary Figure 5. Depletion of PI(4,5)P_2 decreases the plasma membrane cholesterol

a, b Detection of cholesterol using filipin in GFP-, Lyn-INPP5Ewt-GFP (INPP5E WT)-, and Lyn-INPP5Emt-GFP (INPP5E MT)-expressing HaCaT cells (a). Quantification of the plasma membrane (PM) cholesterol signals (b). In total, 103 GFP-, 105 INPP5E WT-, and 114 INPP5E MT-expressing cells were examined over two independent experiments (b). **c, d** Detection of cholesterol using filipin in epithelial (HaCaT and NMuMG) and non-epithelial (HDF, Swiss3T3, and U2OS) cell lines (c). Quantification of the PM cholesterol signals (d). In total, 140 HaCaT cells, 111 NMuMG cells, 107 HDF, 113

Swiss3T3, 107 U2OS cells were examined over two independent experiments (d). ***p = 0.0007, **p = 0.0067 (versus NMuMG cells). **e, f** Detection of PI(4,5)P_2 and cholesterol (filipin) in HaCaT cells treated with TGFβ1 at the indicated time points (e). Images of PI(4,5)P_2 and cholesterol were taken from different samples. Quantification of apicolateral PM PI(4,5)P_2 signals and PM cholesterol signals (f). In total, 103 cells at 0h, 113 cells at 4h, and 111 cells at 48h were examined over two independent experiments for PI(4,5)P_2. In total, 101 cells at 0h, 122 cells at 4h, and 117 cells at 48h were examined over two independent experiments for cholesterol (f). **g** RT-PCR analysis of *OSBPL2* and *GAPDH* mRNA expression in HaCaT cells treated with scrambled (Scr) or *OSBPL2*-targeting (#1 and #2) siRNAs (g). Results shown are representative of two independent experiments. **h** Detection of cholesterol using recombinant mCherry-D4 protein (D4), E-cadherin (E-cad), β-catenin (β-cat), claudin-1, and PARD3 in HaCaT cells treated with scrambled (Scr) or *OSBPL2*-targeting (OSBPL2#1 and OSBPL2#2) siRNAs. Images were taken from different fields of view. **i** Quantification of PM signals of cholesterol, E-cadherin, β-catenin, claudin-1, and PARD3. In total, 123 Scr, 116 OSBPL2#1, and 107 OSBPL2#2 cells were examined over two independent experiments for cholesterol. In total, 103 Scr, 109 OSBPL2#1, and 108 OSBPL2#2 cells were examined over two independent experiments for E-cadherin. In total, 120 Scr, 122 OSBPL2#1, and 110 OSBPL2#2 cells were examined over two independent experiments for β-catenin. In total, 105 Scr, 119 OSBPL2#1, and 118 OSBPL2#2 cells were examined over two independent experiments for claudin-1. In total, 104 Scr, 120 OSBPL2#1, and 118 OSBPL2#2 cells were examined over two independent experiments for PARD3. The box plots are presented with the elements: center line, median; box limits, Q1 and Q3; whiskers, 1.5× interquartile range. Outliers are also shown. Individual data points are displayed (gray points, data from the first experiments; black points, data from the second experiments). Significance was tested using one-way ANOVA with Tukey-Kramer's post hoc test. ****$p < 0.001$ [versus GFP-expressing cells (b), HaCaT cells or NMuMG cells (d), 0h (f), HaCaT cells treated with scrambled siRNA (i)]. Scale bar = 30 μm (a, c, e, h). Source data are provided as a Source Data file.

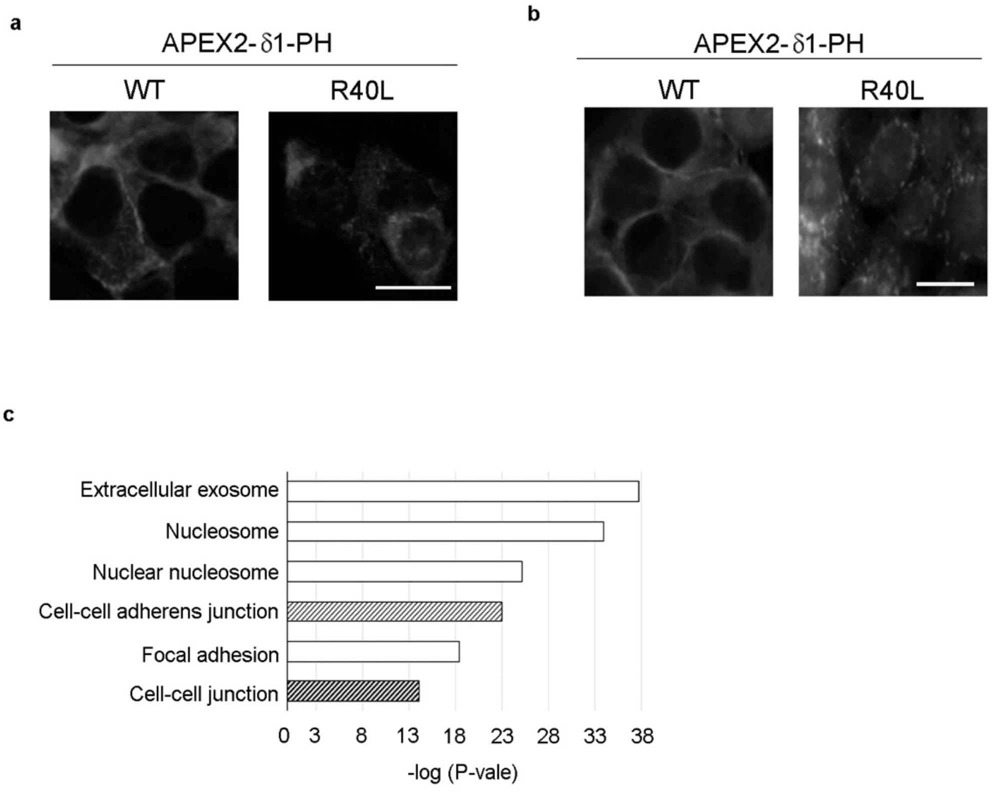

Supplementary Figure 6. Epithelial junctional proteins are PI(4,5)P_2 proximal proteins

a Immunofluorescence detection of the PH domain (WT) or its R40L mutant (R40L) of PLCδ1 fused with APEX2 and V5 (APEX2-δ1-PH) in HaCaT cells. **b** Detection of biotinylated proteins with fluorescent streptavidin in HaCaT cells expressing WT or R40L of APEX2-δ1-PH. **c** Biological pathway analysis of genes encoding putative PI(4,5)P_2 proximal proteins. Results shown are representative of two independent experiments (a and b). Scale bar = 20 μm (a, b). Source data are provided as a Source Data file.

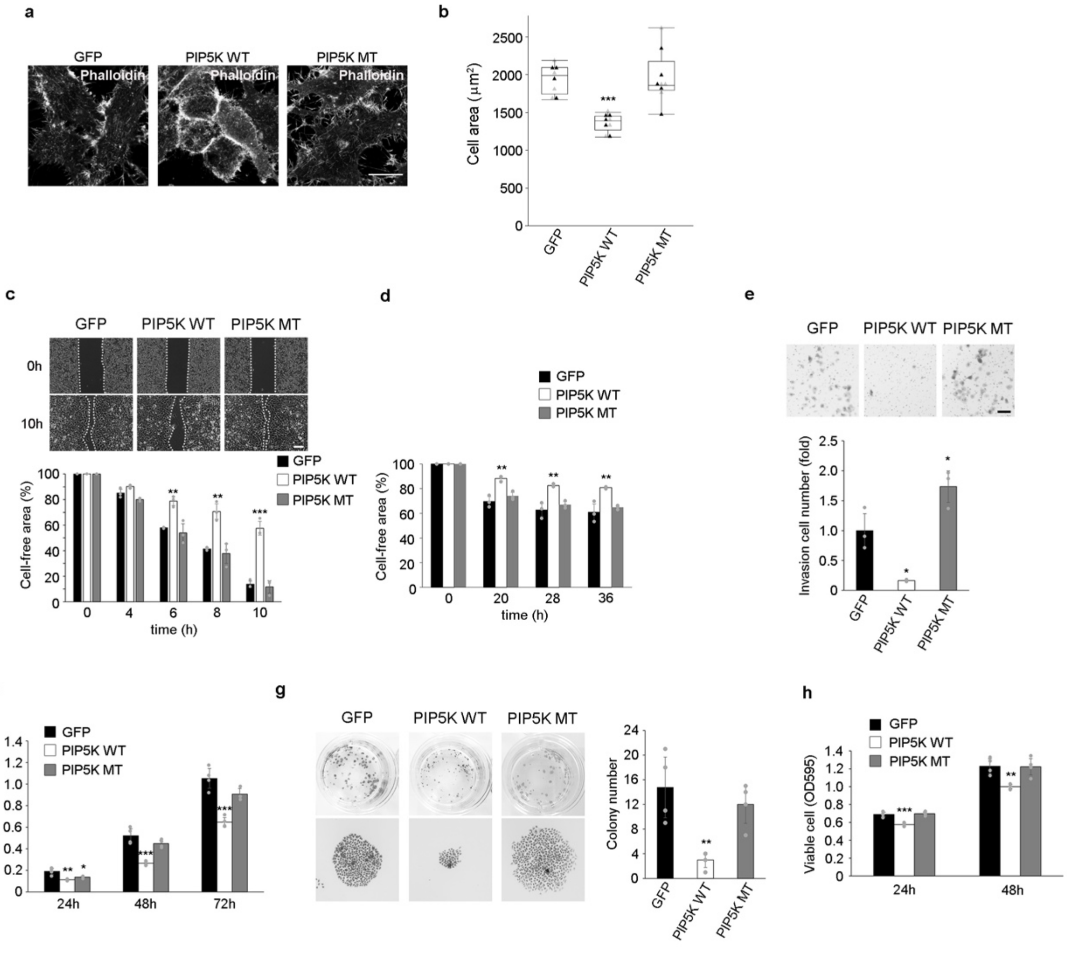

Supplementary Figure 7. Elevation of PI(4,5)P_2 level suppresses aggressive cellular phenotypes of osteosarcoma cells *in vitro*

a Detection of F-actin (phalloidin) in GFP-, Lyn-mPIP5Kwt-GFP (PIP5K WT)-, or Lyn-mPIP5Kmt-GFP (PIP5K MT)-expressing MG-63 cells. **b** Cell area was examined in GFP-, PIP5K WT-, or PIP5K MT-expressing MG-63 cells. Data are represented as mean ± SD. The average cell area was calculated by analyzing eight (GFP-expressing cells) or nine (PIP5K WT- and PIP5K MT-expressing cells) distinct fields of view over two independent experiments. ***p = 0.0001 (versus GFP-expressing cells). The box plots are presented with the elements: center line, median; box limits, Q1 and Q3; whiskers, 1.5× interquartile range; points, outliers. **c-h** Migration (c, d), matrigel invasion (e), proliferation (f, h), and colony formation (g) assays of GFP-, PIP5K WT-, and PIP5K MT-expressing U2OS (c, e, f, g) and MG-63 cells (d, h). The cell-free area was calculated at the indicated time points (c and d). The number of invaded cells was counted using crystal violet staining (e). Number of colonies with more than 50 cells were counted (g). Data are represented as mean ± SD. N = 3 for each group (c, d, e). N = 4 for each group

(f-h). ***p = 0.0002 (c), 0.0001 (f, 48 h), 0.0002 (f, 72h), 0.0002 (h), **p = 0.0093 (c, 6 h), 0.0052 (c, 8h), 0.0018 (d, 20 h), 0.0026 (d, 28 h), 0.0062 (d, 36h), 0.0013 (f), 0.0058 (g), 0.0093 (h), *p = 0.022 (e, PIP5K WT), 0.037 (e, PIP5K MT), 0.0132 (f) (versus GFP-expressing cells). Individual data points are displayed [gray points, data from the first experiments; black points, data from the second experiments (b)]. Significance was tested using one-way ANOVA with Tukey-Kramer's post hoc test. Scale bar = 20 μm (a) and 200 μm (c, e). Source data are provided as a Source Data file.

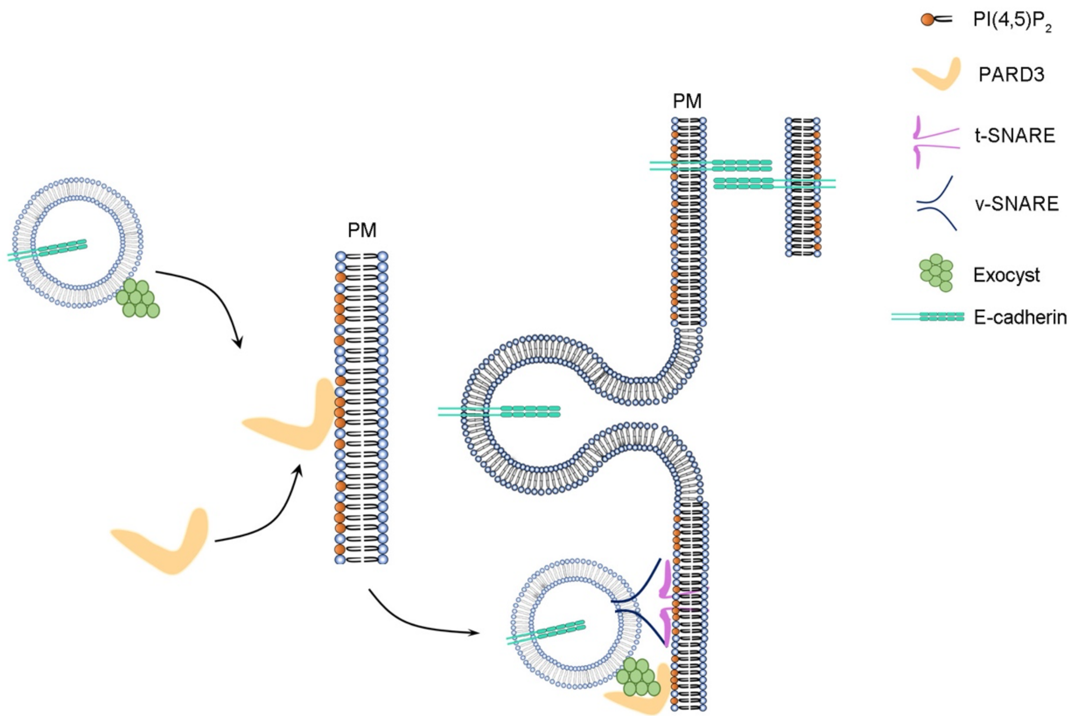

Supplementary Figure 8. The proposed model of $PI(4,5)P_2$-mediated regulation of epithelial characteristics

In epithelial cells, PARD3 and t-SNAREs are recruited to the $PI(4,5)P_2$-rich region of the plasma membrane (PM). PARD3 functions as a receptor for exocysts and recruits exocyst and cargo molecules in vesicles, such as E-cadherin, to the $PI(4,5)P_2$-rich region of PM, leading to the assembly of adherens junctions and the determination and maintenance of epithelial characteristics.

Supplementary Table

Supplementary Table 1. List of Primers

Human *RhoB*	5’-GACTACCTCGAGTGCTCTGC-3’<br>5’ - CAGTTGATGCAGCCGTTCTG-3’
Human *WNT5B*	5’-CGTGGAGTACGGCTACCGCT-3’<br>5’-CAGGCTACGTCTGCCATCTTAT-3’
Human *TAGLN*	5’-GGTGGAGTGGATCATAGTGC-3’<br>5’-ATGTCAGTCTTGATGACCCCA-3’
Human *TNC*	5’-TAGTGGTCAAGTGGGAGGGG-3’<br>5’-GCCTGTAAGCTTTTCCCAAGT-3’
Human *MYL9*	5’-CTGCTTCTGGTCTGTGTGTCT-3’<br>5’-GAAGGTGGGAGAGGGTTTCAG-3’
Human *MYL7*	5’-GAGTGCCTTCCGCATGTTTG-3’<br>5’-AACATCTGCTCCACCTCAGC-3’
Human *JUN*	5’-GAGCTGGAGCGCCTGATAAT-3’<br>5’-CCCTCCTGCTCATCTGTCAC-3’
Human *COL5A1*	5’-GATGTCGCTTACAGAGTCACCAA-3’<br>5’-AAATGCAGACGCAGGGTACAG-3’
Human *FN1*	5’-CCACCACCAGCACCAGCACA-3’<br>5’-ACCCGGAATCCCGACACGGT-3’
Human *CDH2*	5’-GCTCCAAGCACCCCTTCACCC-3’<br>5’-GATGACGGCCGTGGCTGTGT-3’
Human *SNAI1*	5’-CTGCGGGAAGGCCTTCTCT-3’<br>5’-CGCCTGGCACTGGTACTTCTT-3’
Human *SNAI2*	5’-CGGACCCACACATTACCTTGTGTTT-3’<br>5’-CACAGCAGCCAGATTCCTCATGTTT-3’
Human *PIP5K1A*	5’-GATCCCGCGGTCCCTTCCTGT-3’<br>5’-GCCAGAGGCATAAGGCACCAATGA-3’
Human *PIP5K1B*	5’-AGCAGCCTTGATGAAGAAGC-3’<br>5’-GAAGAAGATGAAATTGTGGTTGC-3’
Human *PIP5K1C*	5’-AGCCACTACAGCCTCCATTG-3’<br>5’-TGCCATCCTGTCCAGACGA-3’
Human *OSBPL2*	5’-GCCAATCGCCTTCAACGAG -3’<br>5’-CCACAGCCGAAACAGCAAAAG -3’
Human *ACTB*	5’-CACCCTGAAGTACCCCATCG-3’<br>5’-TAGCAACGTACATGGCTGGG-3’
Human *GAPDH*	5’-GTCTCCTCTGACTTCAACAGCG -3’<br>5’-ACCACCCTGTTGCTGTAGCCAA -3’
