# Supplementary material for: Plasma membrane phosphatidylinositol (4,5)-bisphosphate is critical for determination of epithelial characteristics
Source: Nat Commun. 2022 May 9;13:2347. doi: 10.1038/s41467-022-30061-9 (PMC9085759; doi:10.1038/s41467-022-30061-9)
Supplement: Supplementary file 3 — Description of Additional Supplementary Files [file 41467_2022_30061_MOESM3_ESM.pdf]

Description of Additional Supplementary Files

File Name: Supplementary Data 1
Description: $PI(4,5)P_2$ proximal protein-encoding genes

File Name: Supplementary Data 2
Description: MRM transition and collision energy to detect phosphoinositide variants
