# Supplementary material for: Plasma membrane phosphatidylinositol (4,5)-bisphosphate is critical for determination of epithelial characteristics
Source: Nat Commun. 2022 May 9;13:2347. doi: 10.1038/s41467-022-30061-9 (PMC9085759; doi:10.1038/s41467-022-30061-9)
Supplement: Supplementary file 6 — Reporting Summary [file 41467_2022_30061_MOESM6_ESM.pdf]

nature research

Corresponding author(s): Yoshikazu Nakamura and Kiyoko Fukami

Last updated by author(s): Apr 6, 2022

Reporting Summary

Nature Research wishes to improve the reproducibility of the work that we publish. This form provides structure for consistency and transparency in reporting. For further information on Nature Research policies, see our Editorial Policies and the Editorial Policy Checklist.

Statistics

For all statistical analyses, confirm that the following items are present in the figure legend, table legend, main text, or Methods section.

n/a	Confirmed	
☐	☒	The exact sample size (*n*) for each experimental group/condition, given as a discrete number and unit of measurement
☐	☒	A statement on whether measurements were taken from distinct samples or whether the same sample was measured repeatedly
☐	☒	The statistical test(s) used AND whether they are one- or two-sided<br>*Only common tests should be described solely by name; describe more complex techniques in the Methods section.*
☒	☐	A description of all covariates tested
☐	☒	A description of any assumptions or corrections, such as tests of normality and adjustment for multiple comparisons
☐	☒	A full description of the statistical parameters including central tendency (e.g. means) or other basic estimates (e.g. regression coefficient) AND variation (e.g. standard deviation) or associated estimates of uncertainty (e.g. confidence intervals)
☐	☒	For null hypothesis testing, the test statistic (e.g. *F*, *t*, *r*) with confidence intervals, effect sizes, degrees of freedom and *P* value noted<br>*Give P values as exact values whenever suitable.*
☒	☐	For Bayesian analysis, information on the choice of priors and Markov chain Monte Carlo settings
☒	☐	For hierarchical and complex designs, identification of the appropriate level for tests and full reporting of outcomes
☒	☐	Estimates of effect sizes (e.g. Cohen's *d*, Pearson's *r*), indicating how they were calculated

Our web collection on statistics for biologists contains articles on many of the points above.

Software and code

Policy information about availability of computer code

Data collection	Zen Blue 3.2 software (Carl Zeiss), Zen Black 2.3 software (Carl Zeiss), CFX96 Touch real-time PCR detection system (BioRad), Zeiss LSM900 (Carl Zeiss), BZ-X800 Analyzer (KEYENCE),SH-9000 microplate reader (Corona Electric), MultiQuant 3.0.2 software (Sciex), Analyst 1.6.3 (SCIEX)
Data analysis	Zen Blue 3.2 software (Carl Zeiss), Zen Black 2.3 software (Carl Zeiss), BZ-X800 Analyzer (KEYENCE), ImageJ2 software 2.3.0/1.53f (NIH), JMP Pro14, Mult Quant software (SCIEX), MaxQuant software 1.6.2.3

For manuscripts utilizing custom algorithms or software that are central to the research but not yet described in published literature, software must be made available to editors and reviewers. We strongly encourage code deposition in a community repository (e.g. GitHub). See the Nature Research guidelines for submitting code & software for further information.

Data

Policy information about availability of data

All manuscripts must include a data availability statement. This statement should provide the following information, where applicable:
- Accession codes, unique identifiers, or web links for publicly available datasets
- A list of figures that have associated raw data
- A description of any restrictions on data availability

The mass spectrometry data generated in this study have been deposited in the ProteomeXchange Consortium via the PRIDE partner repository with the dataset identifier the PXD031266. Source data are provided with this paper.

Field-specific reporting

Please select the one below that is the best fit for your research. If you are not sure, read the appropriate sections before making your selection.

[x] Life sciences   [ ] Behavioural & social sciences   [ ] Ecological, evolutionary & environmental sciences

For a reference copy of the document with all sections, see nature.com/documents/nr-reporting-summary-flat.pdf

Life sciences study design

All studies must disclose on these points even when the disclosure is negative.

Sample size	No statistical method was used to define the sample size. The sample size was decided in advance on the basis of common practice of the described experiments. Since the addition of data points did not significantly change the variance of data, sample sizes were sufficient.
Data exclusions	No samples were excluded from the analysis.
Replication	Experiments were repeated at two or three times independently to ensure the reproducibility. Proper statistical analyses were applied to illustrate significance when it's needed.
Randomization	Cells were cultured under the same conditions and randomly allocated into experimental groups.
Blinding	Same researcher performed the experiments and analyzed data, therefore, it was not blinding.

Reporting for specific materials, systems and methods

We require information from authors about some types of materials, experimental systems and methods used in many studies. Here, indicate whether each material, system or method listed is relevant to your study. If you are not sure if a list item applies to your research, read the appropriate section before selecting a response.

Materials & experimental systems

n/a	Involved in the study	
[ ]	[x]	Antibodies
[ ]	[x]	Eukaryotic cell lines
[x]	[ ]	Palaeontology and archaeology
[ ]	[x]	Animals and other organisms
[x]	[ ]	Human research participants
[x]	[ ]	Clinical data
[x]	[ ]	Dual use research of concern

Methods

n/a	Involved in the study	
[x]	[ ]	ChIP-seq
[x]	[ ]	Flow cytometry
[x]	[ ]	MRI-based neuroimaging

Antibodies

Antibodies used

1.Mouse monoclonal anti-GAPDH (clone 6C5) Santa Cruz Biotechnology Cat#sc-32233(1:2000 for immunoblotting)
2.Mouse monoclonal anti-E-cadherin (clone 36/E-Cadherin) BD Transduction Laboratories Cat#610182(1:2000 for immunostaining)
3.Mouse monoclonal anti-E-cadherin (clone 67A4) BioLegend Cat#324102 (1:1000 for immunoblotting)
4.Mouse monoclonal anti-β-catenin (clone 15B8) BioLegend Cat#862602 (1:1000 for immunoblotting; 1:100 for immunostaining)
5.Mouse monoclonal anti-PI(4,5)P2 (clone KT10) In house N/A (3ug/ml for immunostaining)
6.Mouse monoclonal anti-β-actin (clone AC-15) Sigma-Aldrich Cat#A5441 (1:3000 for immunoblotting)
7.Mouse monoclonal anti-N-cadherin (clone 32/N-Cadherin) BD Biosciences Cat#610920 (1:2000 for immunoblotting; 1:100 immunostaining)
8.Mouse monoclonal anti-Claudin1 (clone XX7) Santa Cruz Biotechnology Cat# sc-81796 (1:100 for immunostaining)
9:Mouse monoclonal anti-Claudin1 (clone A-9) Santa Cruz Biotechnology Cat# sc-166338 (1:1000 for immunoblotting)
10. Rabbit polyclonal anti-Par3 Merck Millipore Cat#07-330 (1:200 for immunostaining; 1:2000 for immunoblotting)
11. Rabbit polyclonal anti-V5 BioLegend Cat#903802 (1:2000 for immunoblotting; 1:100 for immunostaining)
12. Rabbit polyclonal anti-PLC delta1 Santa Cruz Biotechnology Cat#sc-30062 (1:500 for immunoblotting)
13. Rabbit polyclonal anti-Akt Cell Signaling Technology Cat#9272 (1:1000 for immunoblotting)
14. Rabbit monoclonal anti-phospho-Akt (Ser473) (D9E) Cell Signaling Technology Cat#4060 (1:1000 for immunoblotting)

Validation

1. Anti-GAPDH (clone 6C5) (#sc-32233; species reactivity, Human; application, immunoblotting) was validated by over 3000 refs as reported by the manufacturer (https://www.scbt.com/p/gapdh-antibody-6c5?productCanUrl=gapdh-antibody-6c5&_requestid=9729614).
2. Anti-E-cadheirn (clone 36/E-Cadherin) (#610182; species reactivity, Human; application, immunostaining) was validated by 5 refs as reported by the manufacturer (https://www.bdbiosciences.com/en-eu/products/reagents/microscopy-imaging-reagents/immunofluorescence-reagents/purified-mouse-anti-e-cadherin.610182).

3. Anti-E-cadheirn (clone 67A4) (#324102; species reactivity, Human; application, immunoblotting) was validated by 3 refs as reported by the manufacturer (https://www.biolegend.com/ja-jp/products/purified-anti-human-cd324-e-cadherin-antibody-3749?GroupID=BLG5130).
4. Anti-β-catenin (clone 15B8) (#862602; species reactivity, Human; ; application, immunoblotting and immunostaining) was validated by 3 refs as reported by the manufacturer (https://www.biolegend.com/ja-jp/search-results/purified-anti-beta-catenin-1-antibody-17559?GroupID=GROUP32).
5. Anti-PI(4,5)P2 (clone KT10) (In house; species reactivity, Human; ; application, immunostaining) was validated (Yamaguchi et al. Cancer Sci. and Yoneda et al. Biochem Biophys Res Commun). This antibody is also sold by abcam (#ab2335) (https://www.abcam.com/pip2-antibody-kt10-ab2335.html) and Enzo Life Sciences (https://www.enzolifesciences.com/ADI-915-062/pip2-monoclonal-antibody-kt10/).
6. Anti-β-actin (clone AC-15) (#A5441; species reactivity, Human; ; application, immunoblotting) was validated by over 8000 refs as reported by the manufacturer (https://www.sigmaaldrich.com/JP/en/product/sigma/a5441).
7. Anti-N-cadheirn (clone 32/N-Cadherin) (#610920; species reactivity, Human; ; application, immunoblotting and immunostaining) was validated by 2 refs as reported by the manufacturer (https://www.bdbiosciences.com/en-us/products/reagents/microscopy-imaging-reagents/immunofluorescence-reagents/purified-mouse-anti-n-cadherin.610920).
8. Anti-Claudin-1 (clone XX7) (#sc-81796; species reactivity, Human; ; application, immunostaining) was validated by 21 refs as reported by the manufacturer (https://www.scbt.com/p/claudin-1-antibody-xx7?productCanUrl=claudin-1-antibody-xx7&_requestid=9958830).
9. Anti-Claudin-1 (clone A-9) (#sc-166338; species reactivity, Human; ; application, immunoblotting) was validated by 44 refs as reported by the manufacturer (https://www.scbt.com/p/claudin-1-antibody-a-9?requestFrom=search).
10. Anti-Par3 (#07-330; species reactivity, Human; ; application, immunoblotting and immunostaining) was validated by over 50 refs as reported by the manufacturer (https://www.merckmillipore.com/JP/en/product/Anti-Partitioning-defective-3-Antibody,MM_NF-07-330).
11. Anti-V5 (#903802; application, immunoblotting and immunostaining) was validated by one ref as reported by the manufacturer (https://www.biolegend.com/ja-jp/products/purified-anti-v5-epitope-tag-antibody-11593).
12. Anti-PLCdelta1 (#sc-30062; species reactivity, Human; application, immunoblotting) was validated by three refs as reported by the manufacturer (https://www.scbt.com/p/plc-delta1-antibody-h-140).
13. Anti-Akt (#9272; species reactivity, Human; application, immunoblotting) was validated by over 2000 refs as reported by the manufacturer (https://en.cellsignal.jp/products/primary-antibodies/akt-antibody/9272).
14. Anti-phospho-Akt (Ser473) (clone D9E) (#4060; species reactivity, Human; application, immunoblotting) was validated by over 2000 refs as reported by the manufacturer (https://en.cellsignal.jp/products/primary-antibodies/phospho-akt-ser473-d9e-xp-rabbit-mab/4060).

Eukaryotic cell lines

Policy information about cell lines

Cell line source(s)	U2OS and NMuMG cells were from ATCC. Swiss3T3 and MG-63 were from JCRB. (JCRB9019), HDF and HaCaT cells were from Kurabo (KF-4009) and Cell Lines Service (300493-ACADEMIC), respectively.
Authentication	Cell line authentication was not performed.
Mycoplasma contamination	Cell lines used were not tested for mycoplasma contamination.
Commonly misidentified lines (See ICLAC register)	No commonly misidentified cell lines used.

Animals and other organisms

Policy information about studies involving animals; ARRIVE guidelines recommended for reporting animal research

Laboratory animals	C57BL/6J mice were purchased from CLEA Japan (Tokyo, Japan). Newborn mice were used for experiments.
Wild animals	This study did not involve the use of wild animals.
Field-collected samples	This study did not involve the use of field-collected samples.
Ethics oversight	All animal studies were approved by the Animal Experiments Review Board of the Tokyo University of Pharmacy and Life Sciences.

Note that full information on the approval of the study protocol must also be provided in the manuscript.
